# Biotechnology landscape in cancer drug discovery

**DOI:** 10.4155/fso.15.10

**Published:** 2015-11-01

**Authors:** Monica Neagu, Radu Albulescu, Cristiana Tanase

**Affiliations:** 1 Immunology Laboratory, “Victor Babes” National Institute of Pathology, 99-101 Splaiul Independentei, 050096, Bucharest, Romania; 2 Biochemistry-Proteomics Laboratory, “Victor Babes” National Institute of Pathology & National Institute for Chemical Pharmaceutical R&D Bucharest; 3 Biochemistry-Proteomics Laboratory, “Victor Babes” National Institute of Pathology & “Titu Maiorescu” University, Faculty of Medicine

**Keywords:** cancer, drug discovery, hits and leads, immune-therapy, microRNA-based technology, next-generation sequencing, signaling pathway arrays, stem cells, technology optimization

Despite all the scientific data gathered in the cancer research domain, this disease remains one of the major death causes worldwide and a heavy societal burden by its associated comorbidities. In this light, drug discovery technologies, their optimization along with the rapid bioinformatics programs development are essential for enlarging cancer therapy approaches [1,2].

Current methodologies for drug development are remotely optimal; as such, we are experiencing an abundance of stage III clinical trial failures. The lack of solid understanding of cancer biology, knowledge that can support the design of confirmatory trials is one of the causes of this failure [3].

Cancer research and drug development have recently entered a new era, along with the concomitant emergence of new technologies, such as whole-genome, proteome profiling or exome sequencing; all these new approaches have led to innovative information. There are several reasons for the discrepancies between the large number of possible future drugs and those approved. US FDA and EMA grant around 20 new molecular drugs entities per year, but in spite of this number there is still a large uncovered area in cancer therapy [3].

The initial research on drug discovery is developed on an academic level, generating all the premises for a certain therapeutic effect obtained upon the inhibition/activation of a protein/pathway/cellular network. Before progression into the lead discovery phase, further validation of the selected target is required [4].

During the last decade, important steps were taken toward the understanding of overall cancer biology and hence specific therapy. Thus, molecular research has revealed major mutations for many cancer types, pinpointing specific targets and, therefore, drug development. Another important step was the comprehension of intratumor cellular pheno/genotype variation that could explain drug's efficacy differences in terms of aiming primary tumors versus metastatic ones in the same patient. An additional significant step was the identification of several processes developed during tumor cells interaction with their microenvironment, process that governs metastasis. Molecular mechanisms of therapy resistance gained knowledge in the drug discovery field [5].

All this recently gathered information converged to the necessity to tailor individualized cancer treatment. The biotechnology landscape confirms that, for each patient, developing gene sequencing, individual protein profiling, intracellular signaling pattern or miRNA signature are paving the road to personalized medicine concept and toward patient-dedicated therapy.

Technology optimization for drug discovery along with sophisticated bioinformatics programs are essentials for optimal cancer therapy. In this view, overlapping biomarkers group and therapy targets clusters seems to be the best approach to design a pool of candidate biomarkers that will serve as major source for potential therapy targets discovery. The most advanced technologies that meet the ‘overlapping criteria’ are next-generation sequencing (NGS), protein/gene array technologies and microRNAs identification as anticancer drugs.

The key preclinical stages in the process of drug discovery were reviewed by Hughes in 2011 [4], and, while the need for optimization covers the whole research and development chain involved in the process of discovery of new drugs, it bears certain distinctive features, due to the aggressiveness and to the diversity of cancer types [6].

Target identification and validation appear as key problems, since appropriate targets have to match several characteristics: to be safe, to be clinically efficacious when targeted, to solve a prior unmet clinical need and, above all, to be easily accessible for the drug's action. After matching these features, upon interaction with the specific drug, the process has to produce a measurable *in vitro* and *in vivo* biological response.

## Next-generation sequencing – innovative complex instrument in drug discovery

The Era that is following the complex project ENCODE (Encyclopedia Of DNA Elements) launched in 2003 has developed high-throughput sequencing technologies and computational strategies. The aim was to evaluate transcriptional process and gene expression in highly complex dynamic molecular and signaling networks. Due to the well-established tumor genomic heterogeneity, NGS technologies empowered clinical new drugs application, tailoring the best target drugs for the each patient [7].

In 2014, a multianalytic panel (MUT-MAP) was developed using next-generation mutation technology. MUT-MAP detected 120 somatic mutations in 11 therapeutic genes [8,9].

NGS-generated data need specific interpretation, hence recently an Integrated Pathway Analysis Database was designed in order to analyze new and existing drug treatment options in particular stratified patients. Therefore, systematically integrating pathway – disease – drug – organ is crucial for understanding the interrelationships between signaling regulatory pathways and drug action. This integration is based on high-throughput omics data (genomics, transcriptomics, proteomics and metabolomics) [10,11]. Recently FDA has approved drugs that aim pathologies triggered by germ line DNA variations, these pathologies being mainly from the oncology domain [12].

Outlining the NGS technology involvement in the biotechnology landscape of cancer drug discovery will lead to unveiling new anticancer drugs.

## MicroRNA-based technology

At the borderline of genomics and proteomics, powerful regulators of cellular activities through post-translational control of protein expression, miRNAs emerged in the last few years, as both disease markers and future drugs. As disease markers, deregulation of miRNAs was found associated to tumorigenesis, initiation and progression in many human cancer types, having versatile functions as oncogenes or tumor suppressors [13]. Individual miRNAs and sets of multiple miRNAs were identified as candidate biomarkers for diagnostics or therapy monitoring.

miRNA expression profiles performed by specific molecular techniques, which include miRNA microarray technology, could improve cancer diagnostic and therapy based on miRNA signature [14–16]. Circulating miRNAs has been found correlated with chemotherapeutical clinical responses, thus identified as efficacy biomarkers for tailored anticancer therapy. As therapy targets, for several miRNAs it has been demonstrated *in vitro* that restoring their normal expression level reduces tumor cells invasiveness or switches them to a ‘normal’ cell phenotype.

New achievements regarding miRNAs involvement in cancer biology define these molecules as excellent candidates for anticancer therapies targets, predicting the outcome or the monitoring therapy efficacy – theranosis [17].

## Technology optimization in hits/leads discovery

The main step in drug discovery research is to identify new molecules (or new exploitation of known molecules) as anticancer drugs. The ‘core’ of hits to leads discovery is represented by the intensive use of high-throughput screening (HTS).

HTS identifies therapeutically effective compounds within molecular libraries (hits), followed by structural optimization and consecutive testing for increased efficacy drug assemblies (leads) to address the molecular/therapeutic target. One important stage consists in the continuous optimization of screening technology (up to the level of ultrahigh throughput technologies) and the intensive use of robotics. In the optimization of this process, bioinformatics and other computational methods are used, aiming to sort-out new hits and leads from different compounds classes [4]. Interactomics represents the main domain that accommodates computational approaches, since there are large databases regarding protein–protein and protein–small molecule interactions. The most relevant cellular interactomics are signaling pathways and networks involved in tumorigenesis.

A considerable advantage is conferred by the possible existence of hits and leads that binds to known domains in a molecule. Molecular modeling, such as docking studies, represents another strategy to define potential agents targeting known targets. This generates the possibility to model the interaction of related (existing or designed) structures. Besides modeling the actual interaction, simulation of efficacy, safety or pharmacokinetics properties simulation and cost-efficacy evaluation can be done.

HTS methods can be cost effective for new drug-molecules discovery; therefore, they can be extended with computational methods, such as virtual screening, which are increasingly important in integrating structural data with more traditional lead optimization techniques [18].

Repurposing of existing drugs also involves the use of *in vitro* screening; this high-throughput approach uses libraries of potential therapeutic agents assayed on multiple cell lines. A literature-based approach – discovery by analogy – can refine the potential drug list and provide a smaller number of active agents aiding the previously mentioned labor intensive method [19].

Reassessment of the correct balance between basic and clinical cancer research aiming toward drug development would have a clear positive outcome in terms of resources and skills dedicated to fundamental cancer research in the era of personalized medicine.

The optimization of drug discovery technologies in cancer achieved by novel methods appears to hold particular promise for strengthening the preclinical valuation of drug candidates.

The application of these technologies should help decision-making regarding drug properties: efficacy, selectivity, mode of action and off-target interactions. This optimal critical path choice would lead to advancement and development of drugs, reducing the inherently high risk associated with drug innovation.

Systematic and consistent integration of biomarker development is necessary for optimized drug delivery based on predicted activity, but also based on patterns of resistance. This emphasizes the need for biological research and biomarker integration to select patients for appropriate therapy, switching from a ‘which patient for this drug’ approach to a ‘which drug for this patient’ approach [3].

Technology optimization for novel cancer drug discovery encompasses high expectation in the benefit of cancer patients. The main guiding principles that aim to accelerate the optimization of technologies include various programs fostering research in drug discovery and accelerating ‘discovery enabling technologies’ progress. Moreover, new multidisciplinary partnerships are mandatory in order to provide patients with an enlarged access to tailored treatments, since the main goal of technology optimization for novel cancer drug discovery is the improvement of cancer patients’ clinical management.

## References

[1] Raghu R, Devaraji V, Leena K (2014). Virtual screening and discovery of novel aurora kinase inhibitors. *Curr. Top. Med. Chem.*.

[2] Tanase CP, Enciu AM, Mihai S, Neagu AI, Calenic B, Cruceru ML (2013). Anti-cancer therapies in high grade gliomas. *Curr. Proteomics*.

[3] Lacombe D, Tejpar S, Salgado R (2014). European perspective for effective cancer drug development. *Nat. Rev. Clin. Oncol.*.

[4] Hughes JP, Rees S, Kalindjian SB, Philpott KL (2011). Principles of early drug discovery. *Br. J. Pharmacol.*.

[5] Yarden Y, Caldas C (2013). Basic cancer research is essential for the success of personalised medicine. *Eur. J. Cancer*.

[6] Janero DR (2014). The future of drug discovery: enabling technologies for enhancing lead characterization and profiling therapeutic potential. *Expert Opin. Drug Discov.*.

[7] Roukos DH (2014). Genome network medicine: innovation to overcome huge challenges in cancer therapy. *Wiley Interdiscip. Rev. Syst. Biol. Med.*.

[8] Schleifman EB, Tam R, Patel R (2014). Next generation mut-map, a high-sensitivity high-throughput microfluidics chip-based mutation analysis panel. *PloS One*.

[9] Schiffman FJ (1986). The teaching house officer. *Yale J. Biol. Med.*.

[10] Zhang F, Drabier R (2012). Ipad: the integrated pathway analysis database for systematic enrichment analysis. *BMC Bioinformatics*.

[11] (1987). Spaet TH. Blood in contact with natural and artificial surfaces. *Ann. NY Acad. Sci.*.

[12] Gillis NK, Patel JN, Innocenti F (2014). Clinical implementation of germ line cancer pharmacogenetic variants during the next-generation sequencing era. *Clin. Pharmacol. Ther.*.

[13] Albulescu R, Neagu M, Albulescu L, Tanase C (2011). Tissular and soluble mirnas for diagnostic and therapy improvement in digestive tract cancers. *Expert Rev. Mol. Diagn.*.

[14] Lynam-Lennon N, Maher SG, Reynolds JV (2009). The roles of microrna in cancer and apoptosis. *Biol. Rev. Camb. Philos. Soc.*.

[15] Zhang B, Pan X, Cobb GP, Anderson TA (2007). Micrornas as oncogenes and tumor suppressors. *Dev. Biol.*.

[16] Adams BD, Kasinski AL, Slack FJ (2014). Aberrant regulation and function of micrornas in cancer. *Curr. Biol.*.

[17] Saumet A, Mathelier A, Lecellier CH (2014). The potential of micrornas in personalized medicine against cancers. *Biomed. Res. Int.*.

[18] Kar S, Roy K (2013). How far can virtual screening take us in drug discovery?. *Expert Opin. Drug Discov.*.

[19] Cohen T, Widdows D, Stephan C (2014). Predicting high-throughput screening results with scalable literature-based discovery methods. *CPT Pharmacometrics Syst. Pharmacol.*.

